# “Fibromyalgia, a life companion that you don’t want to have”: A qualitative study on the impact of receiving a diagnostic label of Fibromyalgia from a patient perspective

**DOI:** 10.1177/13591053251409603

**Published:** 2026-01-26

**Authors:** Mais Tattan, Janna M. Gol, Donald Van Tol, Denise J. C. Hanssen, Judith G. M. Rosmalen

**Affiliations:** 1 University of Groningen, Netherlands

**Keywords:** fibromyalgia, identity, qualitative methods, diagnosis, label

## Abstract

Fibromyalgia is a prevalent condition with significant impacts for patients. This qualitative study aims to explore how patients experience receiving a fibromyalgia diagnostic label. We performed semi-structured interviews with 13 fibromyalgia patients (11 women, 2 men), from six European countries, recruited online through patient organizations. Most of the participants were female and over 40 years old. Interviews were coded and analysed by two independent researchers. Three themes were identified related to receiving the fibromyalgia label: (1) *Changes to myself*, including validation and relief, a perceived threat to identity, and concern for having this condition for life. (2) C*hanges in relation to others*, including losing friends, behaving as part of a group while maintaining individuality of experiences, and developed self-agency. (3) *Changes in social roles*, including altered work status and shifting priorities. We conclude that providing a fibromyalgia label can have multilayered effects on recipients’ personal identity, relationships and social positions.

## Introduction

Fibromyalgia is a condition presenting with characteristic chronic widespread pain, often associated with unrefreshing sleep and cognitive difficulties (Wolfe et al., 2016). The mechanisms and aetiology of fibromyalgia remain largely unknown (Perrot, 2019; Siracusa et al., 2021) despite it being a commonly seen condition with a worldwide prevalence of 1.8% (Heidari et al., 2017). Fibromyalgia is associated with a substantial impact on patients’ quality of life as well as significant economic costs for patients and healthcare systems (D’Onghia et al., 2022; Doebl et al., 2020).

Diagnosis of fibromyalgia is symptom-based and is given according to internationally recognized diagnostic criteria developed by the American College of Rheumatology (ACR; Wolfe et al., 2016). Previous studies have shown that many patients fulfilling the diagnostic criteria for fibromyalgia do not receive a diagnostic label (Tattan et al., 2024; Wolfe et al., 2016). Diagnostic labels are given by healthcare professionals (HCP) to patients depending on the presentations of specific signs and symptoms (Moncrieffe and Eyben, 2007). In addition to facilitating the start of management, diagnostic labels create a space for patients to understand and articulate their own personal identity, influencing their relationships, interactions with HCPs and their position in the community (Sims et al., 2021). Literature shows that this process is not as straightforward for patients with symptom-based diagnoses such as fibromyalgia. Fibromyalgia is a contested and stigmatized diagnosis with many physicians offering different opinions about its validity and legitimacy (Armentor, 2017; Paxman, 2021). Therefore, individuals might consequently experience a denial of legitimacy of their experiences, disempowerment and social isolation (Barker, 2005; Cooper, 1997; Greco, 2024; Ware, 1999).

Previous qualitative studies of patients with fibromyalgia have explored the personal experiences and challenges of living with the symptoms of this condition (Ashe et al., 2017; Boulton, 2019). Such challenges include complex doctor-patient relationships, negative impacts on health journeys, navigating different treatment options, and the need for self-empowerment (Otón et al., 2024; Van Alboom et al., 2025). Moreover, another study found that individuals resist marginalization following a fibromyalgia diagnosis by redefining life goals and actively negotiating their identity (Brown, 2021). However, limited research explored whether receipt of the fibromyalgia diagnostic label leads to specific challenges independently from the challenges of the symptoms of fibromyalgia. In a recent systematic review that studied the impacts of receiving a diagnosis of a functional disorder, including fibromyalgia (Tattan et al., 2025), only few studies investigated the impact of having a diagnostic label of fibromyalgia independently from the impact of the symptoms. In these studies patients reported experiences of stigmatizing encounters after receiving the fibromyalgia diagnostic label (Briones-Vozmediano et al., 2018; Undeland and Malterud, 2007). Other influences, such as impacts on individual identity and social and societal challenges, remain relatively unexplored in relation to the diagnostic label. This qualitative study aims to provide in depth insights into personal experiences of receiving a diagnostic label of fibromyalgia on identity, relationships, and life in general.

## Methods

This qualitative study received ethical approval in collaboration with the University of Sheffield (Ethics application number 046641; 06.05.22). All participants provided written informed consent prior to enrolment in the study. This study employed a phenomenological research approach and followed the Consolidated criteria for reporting qualitative research (COREQ) guidelines (Tong et al., 2007). Phenomenology is an approach that aims to understand how individuals perceive everyday experiences, capturing the essence of these experiences from a person’s unique perspective (Knaack, 1984).

### Sampling and study population

This study used a convenience sample of participants. Recruitment was done in collaboration with Pain Alliance Europe (PAE), which is a non-profit organization based in Belgium, functioning as an umbrella organization of 40 national and regional associations in 17 European countries. PAE aims to promote awareness and reduce the impact of chronic pain across Europe. Information about the study was distributed by PAE and its partner organizations via their online channels, together with a link to an online Expression of Interest form. This form collected participant nationality, languages spoken, main chronic pain condition(s), email address and consent to be contacted about participation in the study. Inclusion criteria for this study consisted of adults (>18) diagnosed with fibromyalgia, and having sufficient English communication skills.

Participants expressing an interest were then contacted by the main researcher by email and a video call was arranged using Microsoft Teams (accessed via University Medical Center Groningen UMCG). In this call, the consent form was explained thoroughly by the main researcher and time was provided for any questions to be clarified. Then the consent form was sent to the participant to sign and return to the main researcher. Informed consent was obtained from all participants prior to the recorded research interview. Participants were allocated a unique study identifier (ID) which served as the only link between personally identifiable data (name, date of birth, address etc.) provided in the consent forms and the study data. All participants who filled the form (*n* = 20) were contacted and only 13 responded back with agreement to participate. Therefore, the study sample consisted of 13 individuals who had received a diagnosis of fibromyalgia. All participants resided in Europe, with four in the UK, three in both Denmark and Sweden, and one in each of Estonia, Slovenia and Ireland.

### Interviews

Interviews were conducted online by the main researcher (MT) in English, in a private room with the presence of only the researcher and the participant interviewed. The researcher introduced herself to the participants as a PhD student and interviews lasted between 60 and 90 minutes. Data collection used open-ended, semi-structured in-depth questions based on an interview guide formulated for this study. The questions in the interview guide were informed by topics identified in a systematic review of quantitative and qualitative studies exploring the impacts of receiving a fibromyalgia label (Tattan et al., 2025) and formulated by the researchers involved in this study. The interview questions were piloted before the official start of interviews with an expert by experience and adjustments were made in content and wording based on the feedback of this patient representative. A summary of the interview topics is provided in Table 1. Data was collected between October 2022 and June 2024 and was stopped when data saturation was achieved (Malterud et al., 2016). All interviews were recorded for analysis and transcription, and no repeat interviews were carried out. Interviews were transcribed verbatim by the main researcher as soon as data was collected in order to include all non-verbal cues that were communicated such as hand gestures or headshakes. Transcribed interviews were not returned to participants for comments or corrections, and no field notes were taken during the interviews. Transcribed interviews were fully anonymized by removing all identifiable information such as name, job description or address by the main researcher.

**Table 1.** Interview guide topics.

Topics discussed
The process of receiving a diagnosis
• General awareness of the fibromyalgia label before receiving it
• Healthcare access and interactions with HCPs
• Communication about the diagnosis
Life before fibromyalgia
• Symptom descriptions
• Decisions to seek help from healthcare
• Interactions with environment about the diagnosis
The name fibromyalgia
• Personal definitions of fibromyalgia
• Identity and fibromyalgia
• Thoughts on the name, advantages and disadvantages
Life with fibromyalgia
• Day to day impacts after receiving the diagnosis
• Changes and reflections on life after receiving the diagnosis

### Analysis

The analysis was carried out by two independent researchers (MT, JG) in Atlas.ti version 24. MT, a female PhD student and medical doctor, and JG, a female psychiatrist with experience in psychosomatic medicine. Besides her clinical work, JG works as a qualitative researcher and is trained and experienced in qualitative analysis. JR, a female researcher in the field of psychosomatic medicine, DH, a female clinical psychologist with experience in psychosomatic medicine, and DvT, a male psychologist experienced in qualitative research, were regularly involved in the discussion of codes and themes and in the interpretation of results. Coding followed an open and inductive approach, where codes were derived from the raw data. This was performed in an iterative process of seeking agreement and adjusting codes. Findings were discussed in regular sessions with the entire research group involved in this study and were not sent to participants for feedback. Data was analysed following the One Sheet Of Paper method (OSOP; Ziebland and McPherson, 2006). This technique involves categorizing the quotes on a single large sized sheet of paper by looking at similarities and differences between the quotes and categories. The different categories were then grouped together under broader themes, with the completed OSOP displaying a full summary of the emerging themes. This process generally captures the themes emerging from the data comprehensively and not simply displaying the most common findings (Ziebland and McPherson, 2006).

Initial plans (in the preregistration) included conducting interviews in other languages, which was not needed as patients from this category did not register for our project. Initial plans also included conducting between 20 and 25 interviews. However, during the data collection phase no new codes were found after the 10th interview, and the decision to stop recruitment after the 13th interview was made based on achieving data saturation and the “information power” concept (Malterud et al., 2016).

## Results

### Study sample

The study’s sample consisted of 13 participants: 11 women and 2 men (Table 2). All participants resided in Europe, with four in the UK, three in both Denmark and Sweden, and one in each of Estonia, Slovenia and Ireland. Most of the participants (*n* = 10) were over 40 years of age and had a long journey towards the diagnosis of over 5 years. Most participants had medical (*n* = 11) and psychiatric comorbidities (*n* = 8). Most participants were full-time workers (*n* = 12) before the diagnosis, while after the diagnosis they were either unemployed or part-time workers (*n* = 10). Over half of the participants received the diagnosis of fibromyalgia from a rheumatologist (*n* = 8) while the rest received it from either a general practitioner (GP), other medical specialist or unspecified physicians. Over half of the participants lived with a partner (*n* = 8) and all of them had high education of at least a bachelor’s degree.

**Table 2.** Demographic data of study participants.

Participant number	Sex	Age range	Country	Time from start of symptoms until diagnosis (years)	Medical comorbidity	Psychiatric comorbidity	Work before diagnosis	Current work	Who gave the diagnosis	Partner presence	Education level
1	Female	40–49	Sweden	>5	Yes	Yes	Full time	Part time	Unspecified physician	Yes	Higher education
2	Female	70–79	Sweden	>5	Yes	Yes	Full time	No	Rheumatologist	Yes	Higher education
3	Female	40–49	Denmark	>5	Yes	No	Full time	Full time	Unspecified physician	No	Higher education
4	Female	20–29	UK	>5	Yes	Yes	No	No	Rheumatologist	Yes	Higher education
5	Female	30–39	Denmark	>5	No	Yes	Full time	No	Pain clinic	Yes	Higher education
6	Female	50–59	UK	>5	No	Yes	Full time	Part time	GP	Yes	Higher education
7	Female	40–49	Denmark	>5	Yes	No	Full time	No	Rheumatologist	No	Higher education
8	Female	50–59	Slovenia	>5	Yes	No	Full time	Full time	Rheumatologist	Yes	Higher education
9	Female	50–59	UK	<5	Yes	No	Full time	No	Immunologist	No	Higher education
10	Female	60–69	Ireland	<5	Yes	Yes	Full time	No	Rheumatologist	Yes	Higher education
11	Male	60–69	UK	<5	Yes	Yes	Full time	No	Rheumatologist	No	Higher education
12	Female	50–59	Sweden	>5	Yes	Yes	Full time	No	Rheumatologist	No	Higher education
13	Male	30–39	Estonia	>5	Yes	No	Full time	Part time	Rheumatologist	Yes	Higher education

### Themes

Data analysis identified three themes outlining changes that occurred to the participants’ self-image, the interaction with their social surroundings, and to their own position in the community after receiving the fibromyalgia diagnostic label. These themes are ***changes to myself***, ***changes in relation to others***, and ***changes in social roles***. Several subthemes emerged under each theme and are elaborated upon using quotes.

#### Changes to myself

This theme describes changes experienced by participants concerning the way they perceive themselves and their own identity. Initially, upon receiving the diagnosis of fibromyalgia, participants expressed emotions of *validation* and *relief* after a long, uncertain and arduous journey to find explanations for the symptoms they were experiencing.

It was a huge relief, it’s one thing to be ill and another thing to not knowing

Participant 12, Female, Age range (50-59)

It was the relief that finally someone had acknowledged the fact that it wasn’t just in my head [. . .] it shouldn’t have taken this long

Participant 4, Female, Age range (20-29)

This *validation* of illness experiences increased participant’s confidence by asserting that the symptoms they suffered from were not imagined but are rather a recognized medical condition.

It was that validation that it wasn’t all in my head [. . .] I had something, somebody gave me that validation that I had a condition and what was happening to me was real.

Participant 6, Female, Age range (50-59)

Nevertheless, the experienced *validation and relief* was short lived and shortly was replaced with many sentiments and concerns. Majority of the participants detailed not only experiencing difficult emotions following the diagnosis but also a perceived *threat to their identity.* This threat stemmed from having to re-discover their priorities and abilities in the present as well as the future.

I felt like I don’t want this, this is not me. This is not my identity. It really triggers your identity, the sense of who you are, because you have to learn who you are all over again [. . .] It made me sad, and it made me mad, and it made me confused, but it also made me happy because something made sense at long last

Participant 7, Female, Age range (40-49)

I think ultimately the initial relief just didn’t last long because [. . .] all the other bits sort of worry about like how is this affecting me in the future? Am I going to struggle if I have a family? Can I have a family?

Participant 4, Female, Age range (20-29)

This *threat to their identity* was also compounded by concerns about being diagnosed with a condition that has no objectively measured proof. This resulted in fears of being disbelieved, experiencing stigma or negative encounters.

It’s difficult to believe that you can have pain without any visible reason [. . .] if you are ok from the outside when nothing is wrong with you, it’s difficult to believe

Participant 8, Female, Age range (50-59)

I realized it’s a really excellent example of an invisible disability and if I had to classify any kind of stigma or any kind of problem I’ve had with people it’s because of that [. . .] because if you look at me, I don’t look disabled [. . .] nobody wants to believe you have a problem, it’s all in your head, I’ve had that told to me

Participant 11, Male, Age range (60-69)

Moving forward, almost all participants in this study mentioned that they believe they would be fibromyalgia *patients forever*. This entailed emotions of grief for the healthy person they once were, and a perceived need for adaptation to a life with limitations.

It’s a process of letting go of the self you’re never going to get back [. . .] I’m never gonna be the person I was when I had energy [. . .] this isn’t like something I’m going to get over and build myself back up from, this is something I have to build myself up with

Participant 5, Female, Age range (30-39)

I stopped fighting it, I realised that it was going to be with me for the rest of my life and either I could let the condition ruin me or I could live my life the way I wanted with the condition

Participant 6, Female, Age range (50-59)

#### Changes in relation to others

This theme illustrates the changes mentioned by participants about their social circles, initially due to developing the symptoms and then after receiving the diagnosis of fibromyalgia. This change affected relationships with close friends, family members, and more distant acquaintances. These interactions shaped the way participants simultaneously communicated about their diagnosis and the way they viewed themselves.

Participants remarked on a process of re-evaluation of existing relationships, specifically when it comes to friends or distant acquaintances. For example, *losing friends* was a topic that came up in almost all of the interviews; some participants attributed this loss to their inability to meet expectations and commented on the vulnerability the diagnosis brings.

It’s very vulnerable because you have to say to people [. . .] I can’t live up to all the expectations anymore. I can’t be that person; I can only do a tiny, tiny bit but I’ll do my best

Participant 7, Female, Age range (40-49)

Other participants commented that *losing friends* could happen as a result of self-protection, which they saw as a process that requires distilling of existing relationships to only include those who accept their current predicament without judgement.

I don’t care as much anymore what people think [. . .] the people who accept me as I am, are the people that I want to stay in my life

Participant 1, Female, Age range (40-49)

As for close friend and family members, almost all participants expressed appreciation for the support and availability of these relationships, for instance *“I’ve been very lucky that way, I have always had good support” Participant 6, Female, Age range (50–59).*

In addition to *losing friends*, participants discussed the challenges of being part of a group diagnosed with the same condition while having different symptoms, experiences and understandings of that condition. Ultimately this divide affected their ability to feel a sense of belonging or unity, which led participants to navigate life with the fibromyalgia diagnosis by being *part of a group but preserving individuality of experiences.*

I think it’s very difficult to feel part of a group because everyone’s experience is different

Participant 9, Female, Age range (50-59)

In talking about feeling part of a group one participant metaphorically described:

A very broad, loosely tied together group, like if we are in a group, the ropes that bind us together are very flappy [. . .] there’s plenty of space because we have the same diagnosis but it’s not the same for anyone

Participant 1, Female, Age range (40-49)

Others observed that while there are differences of experiences within patients with fibromyalgia, having a common diagnostic label extends a sense of comradery and common sympathy between patients and aids in adapting to life with this diagnosis.

There are differences, but we have a lot of commonalities and a lot of struggles that are the same [. . .] it’s just nice to sort of be around people that understand you and you don’t have to explain yourself to because they get it

Participant 5, Female, Age range (30-39)

I think it is useful to have people united, because people within a disease group tend to be very empathetic to each other and would recognise diversity of symptoms and would not be judgmental of others

Participant 10, Female, Age range (60-69)

When asked about the diagnostic label itself, participants remarked that, having a recognized label for their symptoms is useful as it can be used to explain their symptoms to people around them as well as increase their own knowledge about the condition.

It somehow helps mentally, I think, to get a diagnosis, that you can tell other people round and about [. . .] it makes it easier to communicate than just saying I have a headache, or I’m tired all the time

Participant 3, Female, Age range (40-49)

Now I understood because I had a name to it, I can now look things up

Participant 11, Male, Age range (60-69)

In addition to *losing friends* and being *part of a group but preserving individuality of experiences*, a significant change in communication can be seen in patients’ interaction with HCPs. Participants indicated that having a diagnosis of fibromyalgia meant that they were exposed to dismissal and disregard from HCPs. Many participants attributed these negative experiences with HCPs to physicians often considering fibromyalgia as a last resort, judging by the very broad and diverse symptom presentations that can be attributed to the condition.

Right now, it’s like what we describe as a waste bin diagnosis, it’s that if nothing else goes, this is it, and when you probably get less help from doctors because they don’t know what to do with the diagnosis

Participant 13, Male, Age range (30-39)

To assert some control over how they were perceived and which resources they could access, most participants actively choose the situation and the person for whom they communicated having this diagnostic label to. In other words, participants *developed self-agency*, which allowed them to carefully navigate professional and personal interactions, for example, one participant when talking about communicating the diagnosis to HCPs stated:

If I go to an out-of-hours doctor with pain I will not say it unless I feel like because I don’t want them to dismiss me [. . .] I will not lead with saying I have fibromyalgia, I will want them to look at the joint without being coloured by the diagnosis

Participant 5, Female, Age range (30-39)

This hesitancy and meticulous consideration before mentioning the diagnosis was expressed by the majority of participants, not just with HCPs but also in regular personal interactions.

If I meet someone, but if I feel it’s appropriate, I mean depending on the situation [..] If I feel, I’ll mention fibromyalgia quite openly and normally

Participant 6, Female, Age range (50-59)

#### Changes in social roles

This theme shows the changes in social roles that occurred after receiving the diagnostic label. Almost all participants in our study talked about how having the fibromyalgia diagnosis has resulted in an *altered work status* as the majority of the participants transitioned from full-time employment into part-time or unemployment. This transition was not immediate after receiving the diagnosis and happened gradually over many years with many factors contributing to it, such as the intensity of the symptoms and individual and personal choices. Participants who underwent this change talked about the financial impacts of this step alongside feelings of loss and grief for work and worry about what the future holds.

There’s a huge financial impact, I mean, I’ve gone from a very well-paid job in the city to now living off my savings and worrying about how I can afford anything

Participant 9, Female, Age range (50-59)

Whereas the fibromyalgia symptoms might have contributed to the altered work status, the diagnostic label could contribute to some financial stability due to available assistance through social benefits. However, participants also mentioned the difficulty they encountered in obtaining this support.

The diagnosis only helped me with getting support like benefits from the state to cover my rehabilitation expenses [. . .] benefits have actually made my routine and symptoms a lot better, the most difficult part is getting the benefits and support [. . .] a lot of the times you have to go and debate decisions like prove that you actually have issues

Participant 13, Male, Age range (30-39)

I get benefits but since having them and the diagnosis as well, I have had to fight every time I get reviewed

Participant 4, Female, Age range (20-29)

In addition to the *altered work status*, participants reflected on their life with fibromyalgia before and after receiving the diagnosis. Most of them voiced stories of a conscious re-focus to centring important relationships and a choice to pursue *a priority focussed life* after the diagnosis. Some of the participants fulfilled this new life by becoming patient advocates and helping other fibromyalgia patients navigate life with this diagnosis. Others preferred to be more available for their families and play a bigger part in the lives of people they cared about, while some chose to continue their lives uninterrupted by the symptoms and find joy through work.

I miss my work, I have a good work now I think, I very often say I got a new life, it’s not the same as before and there is a lot of sadness over that but I have a good life now [. . .] There is a life to live and you need to be supported and to get help but there is a life to live

Participant 2, Female, Age range (70-79)

If I had to find a silver lining, would be that I have really narrowed down what’s important in my life [. . .] the value of living with a partner like fibromyalgia is finding out the importance of what kind of life I really want to live, because now I’m not capable of choosing from a palette of 500 different things, I might have a palette that has 8 different things, so I have had to narrow my life, the quality of my life, down to a pin

Participant 7, Female, Age range (40-49)

There was a change of mindset [. . .] I think I was focused quite a lot on what I wasn’t doing and what I couldn’t do, so then I was able to focus on what I was managing to get done each day and focus on the small wins, as I call them, rather than looking for bigger ones

Participant 6, Female, Age range (60-69)

### Summary

**Figure 1.**
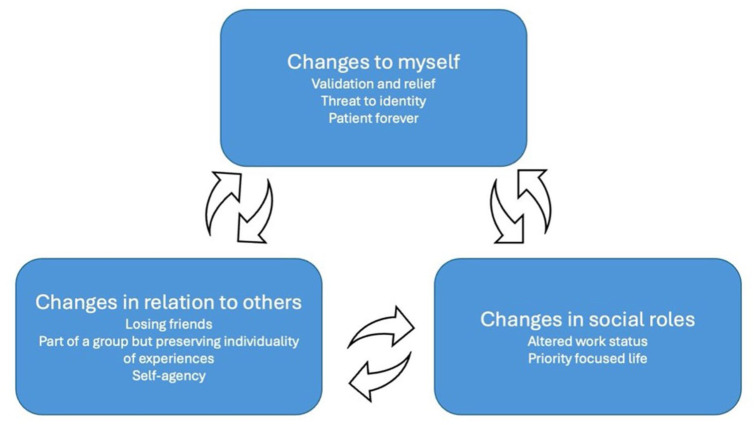
List of themes and subthemes and interactions between them.

Figure 1 summarizes themes and subthemes of this study and the interactions between them. The three themes uncovered in this study illustrate changes perceived by participants after receiving the fibromyalgia diagnosis. The way participants viewed themselves affected their interactions with the social surroundings making it preferable to distill friendships, be a part of a group while preserving individuality of experience, develop self-agency, and alter work status or have a priority focussed life. Similarly, interactions with the social surroundings influenced the way participants viewed themselves, either by validating their experiences, magnifying the threat to their identity (especially through negative encounters), or inspiring a modification of behaviour and life trajectory. Likewise, the alteration of work status and having a priority focussed life affected participants self-image and the way they interacted with their environment.

## Discussion

The findings of this qualitative study reveal that having received a fibromyalgia diagnostic label has effects on several levels. Participants in this study reflected on changes to their self-image, that included validation of their experiences upon receiving the diagnostic label, a perceived threat to their identity after acquiring this label and a voiced concern for having this condition for life. Additionally, participants described a change in relationships with their social surroundings, from losing friends to behaving as part of a group while maintaining individuality of experiences, to developing self-agency. The last change perceived by participants was seen in their position in society, evident in the change in working status or through shifting priorities to focus on loved ones or through helping other patients with fibromyalgia.

Participants’ narratives in this study echoed existing themes found in the literature exploring fibromyalgia patients’ self-image and identity (Mengshoel et al., 2018; Wuytack and Miller, 2011). After receiving the fibromyalgia diagnostic label, participants in this study experience a short-lived validation of experiences and relief, which promptly changed into a perceived threat to identity. This is similar to existing literature: a meta-ethnography concluded that experiences of fleeting initial relief upon receipt of the fibromyalgia diagnosis were reported across many studies (Mengshoel et al., 2018). In regard to identity changes, a previous study found that patients with fibromyalgia face an existential disruption or an identity collapse due to the challenge of living with the symptoms (Wuytack and Miller, 2011). Other studies attributed identity changes to the unpredictability of the illness course (Lempp et al., 2009), or to the chronicity of symptoms and the continued evaluation of everyday tasks in light of the symptoms (Paxman, 2021). Furthermore, the belief of having the condition for life was reported in a recent study focussing on doctor-patient interactions, where patients also shared that having this condition, while not life threatening, remains a reason for concern due to the absence of a cure (Varinen et al., 2022). These insights could offer an explanation to the perceived threat in identity experienced by participants in our study. Additionally, our study adds that having the label itself intensifies perceived identity changes and challenges to self-image as patients also deal with making sense of the diagnostic label itself.

The second theme seen in this study is the changes occurring in relationships and communication. Participants in this study described loss of some relationships and often simultaneous fortification of other ones. This is consistent with existing literature as a re-evaluation of existing relationships was seen in patients with fibromyalgia, where some relationships reportedly became strained while others were strengthened (Crowe et al., 2017; Sim and Madden, 2008). Additionally, receipt of a diagnosis resulted in an automatic inclusion to the collective group of patients with fibromyalgia. This inclusion was met with resistance from this study’s participants as they observed differences of experiences between patients with this condition. This is similar to earlier studies that reported ambivalence towards fibromyalgia support groups and online communities with experiences of support and comradery alongside struggles with unity and inclusion (Ashe et al., 2017; Sallinen et al., 2011). An addition of the current study focussing on the diagnostic label is our finding that participants developed self-agency with regard to explaining their condition. This could be explained by the repeated negative interactions with healthcare professionals and stigmatizing encounters with the surrounding environment (Ashe et al., 2017; Lempp et al., 2009; Paxman, 2021). Additionally, Fibromyalgia patients often experience an imbalanced doctor-patient relationship and a lack of trust in healthcare systems. Our participants’ development of self-agency and selective disclosure of their diagnosis mirrors these findings, suggesting that the label itself influences how patients navigate healthcare and social interactions (Kachaner et al., 2023). Research suggests that specific HCP training is a way to counter stigma and its impacts on patients. Such training should involve people with lived experience and interactive delivery methods, and be followed by an evaluation of behavioural changes in HCP (Guerrero et al., 2024). This could potentially lower the need for patients to cope with stigmatizing encounters and facilitate better communication particularly when interacting with HCPs. As for the social environment, awareness campaigns and more understanding of conditions like fibromyalgia can contribute to reduction of stigma encountered by patients.

The findings of this study shed a light on the changes occurring in occupational status and future outlooks of patients with fibromyalgia. Most of the participants in our study transitioned from full-time employment to none or part-time employment, highlighting symptom effects on work ability. This is in accordance with previous studies that have reported significant impacts of fibromyalgia on occupational status, future perspectives and life paths (Ashe et al., 2017; Briones-Vozmediano et al., 2016; Sallinen and Mengshoel, 2019). However, this study adds that receipt of the label served as a turning point which facilitated the transition in work status for some participants through acceptance of individual limitations or through access to social benefits. A recent systematic review showed that identity and personal characteristics as well as experiences of patients with fibromyalgia greatly influence occupational status in this patient group (Dépelteau et al., 2021). This is consistent with our findings that show interactions between changes in self-image and identity and the changes in social roles in patients suffering from fibromyalgia.

Lastly, a meticulous consideration of priorities was reported by participants in our study. Centring of important relationships, providing guidance to other patients or finding fulfilment through continued work were seen as life strategies for the future. This is in line with previous literature as patients with fibromyalgia emphasized the importance of important relationships such as familial bonds (Wuytack and Miller, 2011). Similar to our findings, a study of Arab patients with fibromyalgia, observed that the diagnostic label shaped patients’ coping strategies and their engagement with peer support, where the label fosters both connexion and differentiation among patients (Aldarwesh, 2025). Furthermore, previous studies also reported that receiving the fibromyalgia label was essential for forming a future outlook and in developing coping strategies for patients with this condition (Arnold et al., 2008; Mengshoel and Heggen, 2004; Wuytack and Miller, 2011).

### Strengths and limitations

This study has some strengths as well as limitations. A strength of this study is the inclusion of a wide range of participants, and from several European countries through online recruitment. The choice of using this method of recruitment was made as the start of this study coincided with the Covid-19 pandemic restrictions, which may have negatively affected availability of participants. Nevertheless, recent studies show that online interviews produce similar data volumes and sharing of personal information as in-person interviews, as well as offering convenience in terms of scheduling and travel arrangements (Krouwel et al., 2019). A limitation of this study is that the recruitment was done via online channels of patient organizations, which might have resulted in selection bias of participants in which fibromyalgia is a relatively important part of their life. Due to the use of convenience sampling, we had limited control over the diversity of the sample, which resulted in a predominance of participants with higher educational backgrounds. In addition, fibromyalgia diagnoses in our participants were made by different healthcare professionals who may have used different criteria, so we cannot be sure that all our participants indeed fulfil standardized diagnostic criteria. However, since this study is on the impact of receiving the fibromyalgia label and not on the aetiology of the disorder, we deem the exact diagnostic criteria used less relevant. Moreover, there is limited representation of European countries in our study with few or only one participant from each country and therefore we cannot provide concrete information on the diverse nature of healthcare systems in each of the represented countries. Furthermore, the inclusion of only English speakers limited our sample and may have affected motivation to participate. Finally, because of convenience sampling and as fibromyalgia predominantly affects females, we encountered low numbers of male participants during recruitment. This could have led to the underrepresentation of male patient experiences with fibromyalgia. Future studies could specifically focus on the differences between the experiences of women and men with fibromyalgia.

## Conclusion

In conclusion, this study highlights the importance of exploring the effect of diagnostic labelling for patients with fibromyalgia apart from the effects of symptom development. Although a diagnostic label might provide initial relief for patients, it might also have consequences for their self-image, relationships with others, and professional lives. This implies that HCPs should balance these potential positive and negative consequences before providing a diagnostic label. This requires a good relationship between patient and HCPs, but this study also shows that there is room for improvement in this regard. Increased physician understanding of the potential implications of receiving a fibromyalgia diagnostic label could contribute to fewer negative or stigmatizing encounters between HCPs and fibromyalgia patients, and eventually better understanding and integration into patient’s identities and life outlooks.
